# Phase synchronization of chlorophyll and total phosphorus oscillations as an indicator of the transformation of a lake ecosystem

**DOI:** 10.1038/s41598-022-16111-8

**Published:** 2022-07-13

**Authors:** Alexey V. Rusakov, Boris V. Adamovich, Nailya I. Nurieva, Raisa Z. Kovalevskaya, Tamara M. Mikheyeva, Nataly P. Radchikova, Hanna A. Zhukava, Yulia K. Veres, Tatyana V. Zhukova, Alexander B. Medvinsky

**Affiliations:** 1 Institute of Theoretical and Experimental Biophysics, Russian Academy of Sciences, Pushchino, 142290 Russia; 2 Belarusian State University, 220030 Minsk, Belarus; 3 Moscow State University of Psychology and Education, Moscow, 127051 Russia

**Keywords:** Ecology, Ecosystem ecology

## Abstract

The ecosystem of the Naroch Lakes (Belarus) includes three water bodies, Lake Batorino, Lake Myastro and Lake Naroch. These lakes have a common catchment area. At the end of the 80 s, the ecosystem of the Naroch Lakes underwent a transformation, during which the nutrient load on the catchment area decreased, and the concentration of phosphorus as a limiting factor in these water bodies decreased significantly. At the same time, the Naroch Lakes were exposed to zebra mussel (*Dreissena polymorpha* Pallas) invasion. In the mid-90 s, the biological and hydrochemical characteristics of the ecosystem of the Naroch Lakes stabilized. We show here that complex processes associated with the transformation of the lake ecosystem and affecting both trophic interactions in the Naroch Lakes and the influence of environmental factors on them can be represented using a single parameter, the phase-locking index, *PLI*. In this case, a statistically significant numerical value of *PLI* characterizes the phase synchronization of two processes, oscillations of the concentration of total phosphorus, TP, and oscillations of the concentration of chlorophyll, Chl. We show that the phase synchronization of these processes occurs only after the stabilization of the ecosystem of the Naroch Lakes. In the period preceding the transformation of the lake ecosystem, there was no synchronization. Numerical evaluation of *PLI* as a holistic parameter allows us to characterize the transformation of the lake ecosystem as a whole without resorting to study of complex interactions of various factors involved in this transformation.

## Introduction

The study of the phosphorus load, as well as the factors determining the dynamics of phosphorus in reservoirs, became of paramount importance in aquatic ecology after it was established in the middle of the twentieth century that phosphorus significantly limits the development of phytoplankton in freshwater reservoirs and, accordingly, determines their potential productivity^1–7^. Despite the long-lasting history of studying the effect of phosphorus on water bodies, a number of issues still remain not completely obvious, and the problems of eutrophication, which worsened in the middle of the twentieth century, remain relevant to the present^7–10^. All this determines the continuing interest in the study of phosphorus dynamics in various types of reservoirs.

Phosphorus exists in different forms in water. It can be dissolved, bound to particles of soil and other materials or contained within living or decaying plants and animals. In addition, phosphorus can enter lakes due to runoff from farmland or fertilized lawns^10^. Total phosphorus (TP) represents all the forms of phosphorus listed above combined. Relationship of phosphorus with chlorophyll (Chl) allows TP to be used to determine lake trophic state^11^.

Chlorophyll *a*, essential for plant photosynthesis, is regularly measured^12,13^ as an indicator of total biomass. The concentration of Chl is one of the key indicators to assessing the productivity and ecological state of aquatic ecosystems^11,14^.

In many lakes, TP is mainly associated with phytoplankton^9,15–19^. As a result, variations in phytoplankton biomass would be associated with changes in both Chl and TP, which may lead to a correlation between the two. However, in addition to phosphorus, nitrogen is sometimes a limiting nutrient for algal growth^7,20–22^. The Chl-TP relationships also depend on a number of other factors, such as trophic interactions in the phytoplankton-zooplankton chain and variations in the concentration of dissolved organic carbon^10^. The combination of these factors can result in a complex, poorly predictable character of variations in the Chl-TP relationship.

Here, we compare the Chl-TP relationships before and after the transformation of the ecosystem of the Naroch Lakes, which occurred as a result of both a biological invasion and a decrease in the nutrient load. As a quantitative characteristic of such a transformation, we used the magnitude of the phase synchronization of Chl and TP oscillations. Numerical estimates of synchronization of oscillatory processes are widely used in the analysis of the dynamics of natural, technical and social systems^23^. In this paper, the phase-locking index (*PLI*)^24^ acts as a synchronization measure. *PLI* has gained widespread application in medical research^25^. At the same time, as to our knowledge, *PLI* has rarely been used to study ecological processes (see^25^ as an example). We show here that the phase-locking index (*PLI*), which characterizes the phase synchronization of irregular Chl and TP oscillations, can be considered as the indicator of the transformation of the lake ecosystem.

## Study site

**Figure 1:**
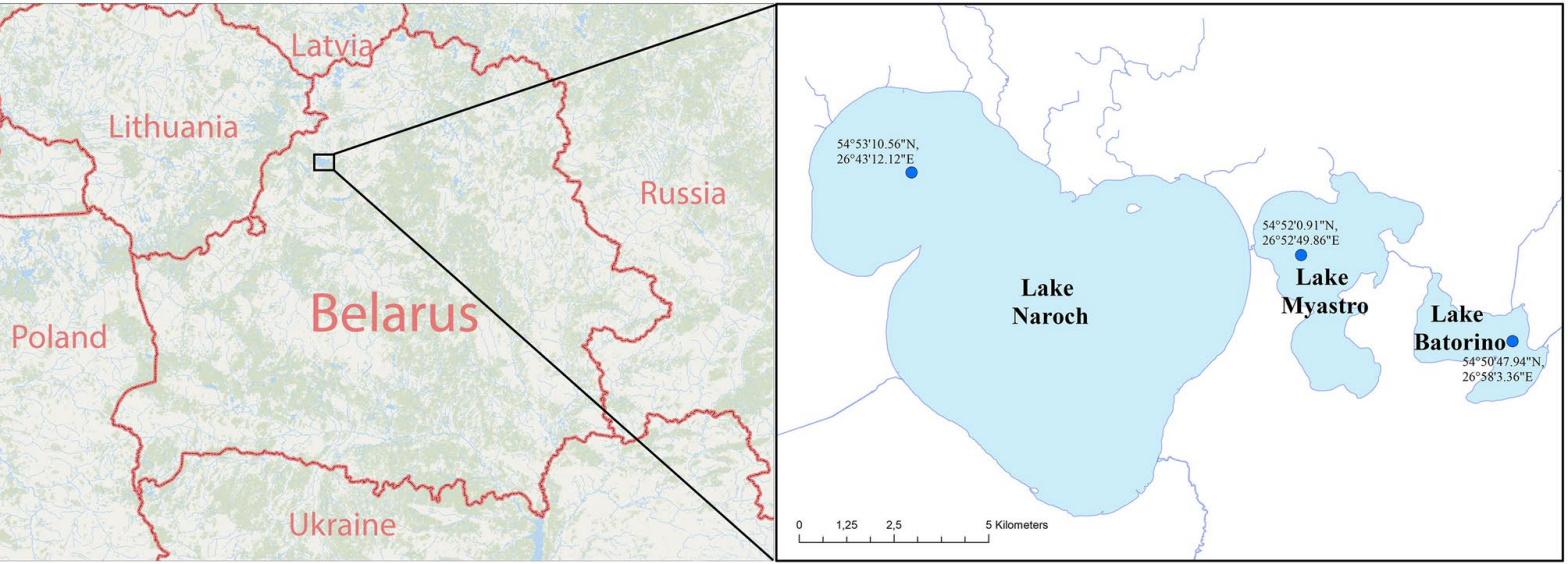
Geographical location of the Naroch Lakes. The map was created using ArcMap 10.6.1 (https://www.arcgis.com/index.html). The coordinates of the sampling sites are marked (see also the “Methods” section).

We investigated the Chl-TP relationships in the Naroch Lake system, which is situated in the Northwestern Belarus (Fig. 1). It includes three lakes that are interconnected by channels, Lake Batorino, Lake Myastro and Lake Naroch, and are all polymictic, i.e., intensively mixed (Table 1).

**Table 1:** The main characteristics of the Naroch Lakes.

Characteristics	Naroch	Myastro	Batorino
Surface area, km^2^	79.6	13.1	6.3
Water volume, mln m^3^	710.0	70.1	18.7
Depth (average/maximum), m	8.9/24.8	5.4/11.3	2.4/5.5
Trophic state	Oligotrophic-mesotrophic	Mesotrophic	Eutrophic
Mixing	Polymictic	Polymictic	Polymictic

The Naroch Lakes were monitored intensively during the last fifty years. In the late 1970s, the increased nutrient load on the catchment area was followed by eutrophication of the Naroch Lakes. Hereafter, two external factors had a significant impact on the ecosystem of the Naroch lakes: (1) implementation since 1981 of the State Program of integrated use and protection of water and land resources of the Lake Naroch basin, which, in fact, was the beginning of the period of re-oligotrophication; (2) the introduction and mass distribution of the water filtering mollusk *Dreissena polymorpha* Pallas in all three water bodies since the early 1990s.

In the early 80 s, a General Scheme for the integrated use and protection of water and land resources of the lake basin was adopted. A number of environmental measures were carried out in the catchment area of the Naroch Lakes. Farmland was repurposed, the water protection zone was planted, the remaining livestock farms were removed from the catchment area and improved, and a bypass collector was built for collecting, subsequent purification and discharge of wastewater outside the Naroch Lakes catchment area. As a result of the measures carried out, the phosphorus load from the catchment area on Lake Naroch decreased by 40–48%, on Lake Myastro by 22–27%, and on Lake Batorino by 34–36%^27^.

In addition, the Naroch Lakes were affected by the invasion of zebra mussel *Dreissena polymorpha* Pallas^28–30^. In Lake Myastro, *Dreissena* was first noted in 1984, and the first *Dreissena* finds in Lake Batorino date back to about the same time^34^. In the following years, there was an abrupt growth of the *Dreissena* population. In 1990, the average biomass of *Dreissena* (± standard error) in Lake Naroch was 1.5 ± 0.6 g/m^2^, and by 1993 was 99 ± 30 g/m^2^. In Lake Myastro, the average biomass of *Dreissena* in 1993 was 402 ± 187 g/m^2^, and in Lake Batorino was 79 ± 13 g/m^2^. Considering that within two years after the first detection in lakes, the abundance of *Dreissena* usually reaches values close to the maximum^28^, it can be assumed that since 1991 in Lake Naroch and a little earlier in Lake Myastro and Lake Batorino, *Dreissena* began to significantly affect ecosystem processes. The stabilization of biological and hydrochemical characteristics of these water bodies came in the mid-1990s^29,31,32^.

More information is available in the “Methods” section.

## Results

**Figure 2:**
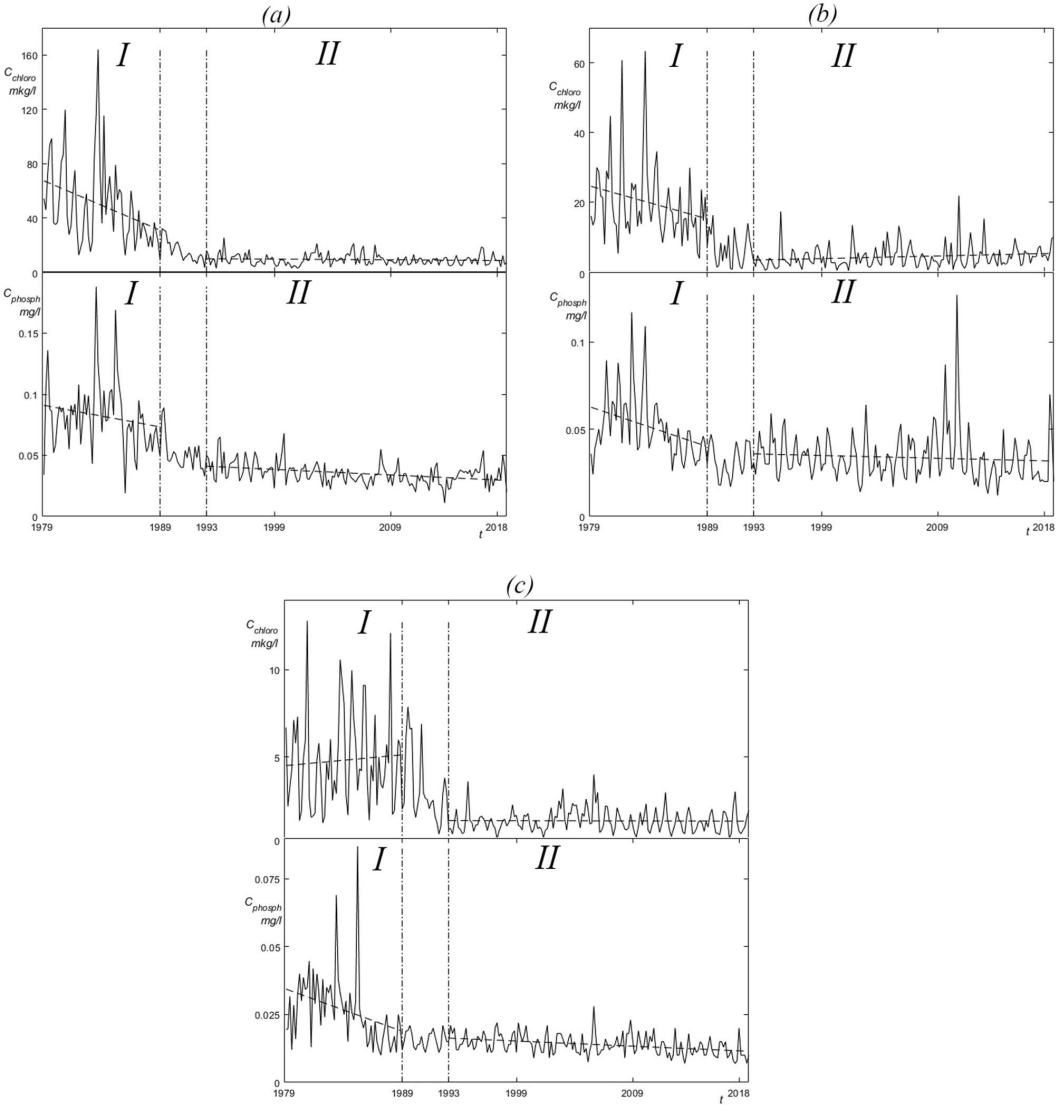
Time series representing the Chl (*C*_chloro_) and TP (C_phosph_) concentration oscillations: (**a**) Lake Batorino, (**b**) Lake Myastro, (**c**) Lake Naroch. Vertical dashed dotted lines show the boundaries of the division of the studied data into the period (*I*) of high anthropogenic load (1979–1988) and the period (*II*) of low anthropogenic load (1993–2018). Short-lived (1989–1992) transients are not analyzed here.

Chl and TP oscillations illustrate the transformation in the Naroch lake system from a period characterized by the high nutrient load (period I) to period characterized with low nutrient load and dreissenids (period II) (Fig. 2). Although the oscillations remain irregular throughout the entire observation period, their amplitude decreased significantly following the transition from period I to period II.

The time series of monthly chlorophyll and total phosphorus were correlated for Lake Myastro and Lake Batorino in both period I and II; however, the Chl oscillations in Lake Naroch had no significant correlation with the TP oscillations for either time period (Table 2). A comparison of these results will be discussed in context of the estimates of the phase synchronization (i.e., the phase-locking index, *PLI*) of the Chl and TP oscillations.

**Table 2:** The Spearman correlation coefficients between Chl and TP concentrations for Lake Batorino, Lake Myastro and Lake Naroch; p < 0.01 (**), p < 0.001 (***).

Water body	1979–1988	1993–2018
Lake Batorino	0.62*** (n = 52)	0.31*** (n = 149)
Lake Myastro	0.38** (n = 54)	0.34*** (n = 151)
Lake Naroch	0.04 (n = 54)	0.15 (n = 151)

**Figure 3:**
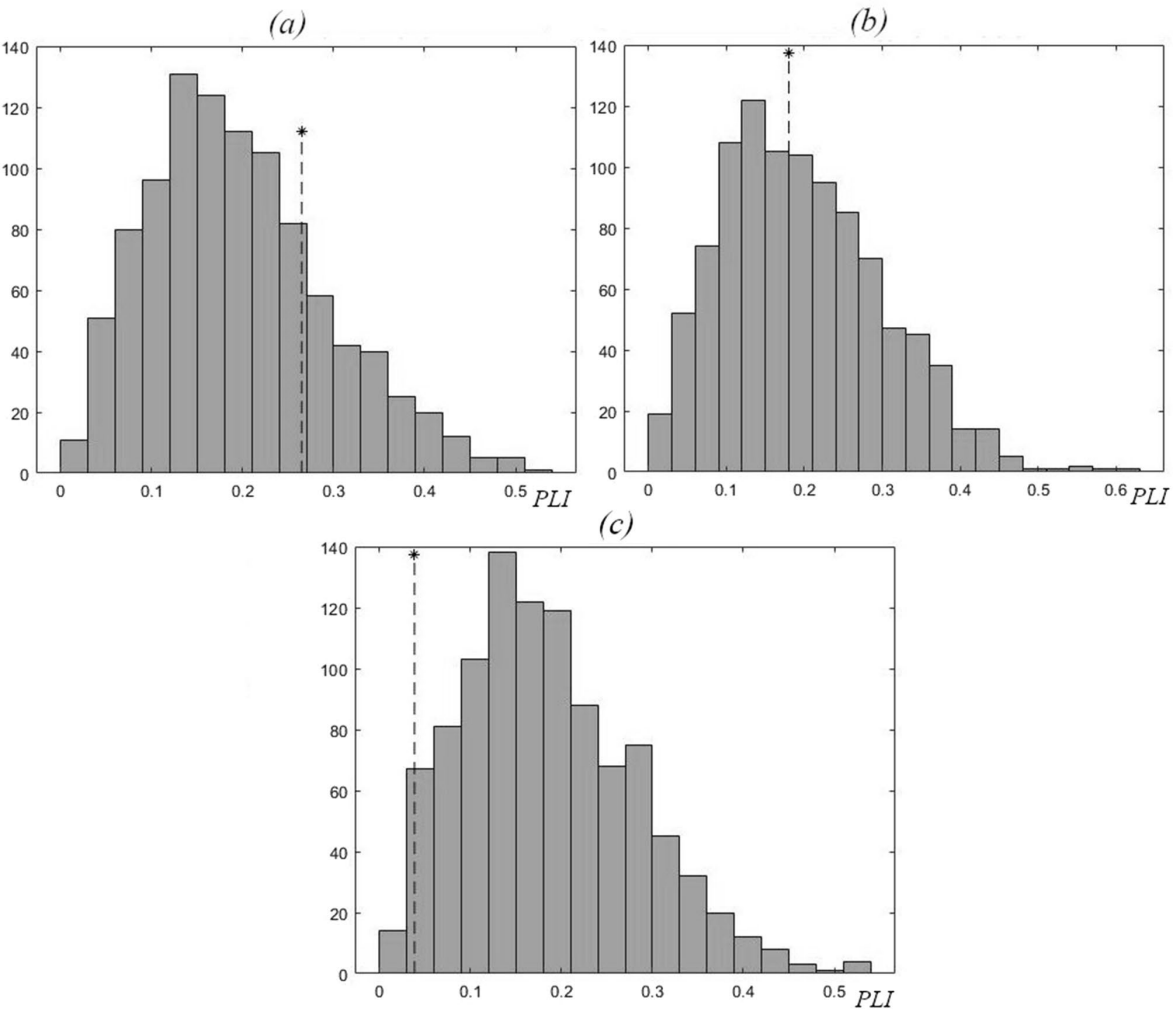
Values of *PLI* (shown by *) for 1979–1989, and the corresponding distributions of *PLI* values for the surrogate data resulted from 1000 random shuffles of the intial Chl and TP time series for each of the Naroch Lakes: (**a**) Lake Batorino (*PLI* = 0.26), (**b**) Lake Myastro (*PLI* = 0.18), (**c**) Lake Naroch (*PLI* = 0.04).

To estimate the phase synchronization of two oscillatory processes, it is necessary to remember that the numerical value of *PLI* can depend on the moment of time (*t*_*n*_) to which the initial phase value equal to 2π*n* (*n* = 0, 1, 2, …) is assigned (see Methods for more details). The values of *PLI* (for 1979–1988) for each of the reservoirs under study are shown in Fig. 3. As can be seen from Fig. 3, the *PLI* values are inside the *PLI* distributions that were obtained during testing using surrogate data, i.e. inside the distributions obtained as a result of random shuffling of the initial Chl and TP time series; the shuffled time series are independent and out of sync^34^. During period I (1979–1988), there was no synchronization detected between the oscillations of Chl and TP concentrations in any of the Naroch system lakes for either moments *t*_*n*_ corresponding to the maximum or minimum values of Chl and TP time series (Fig. 3).

**Figure 4:**
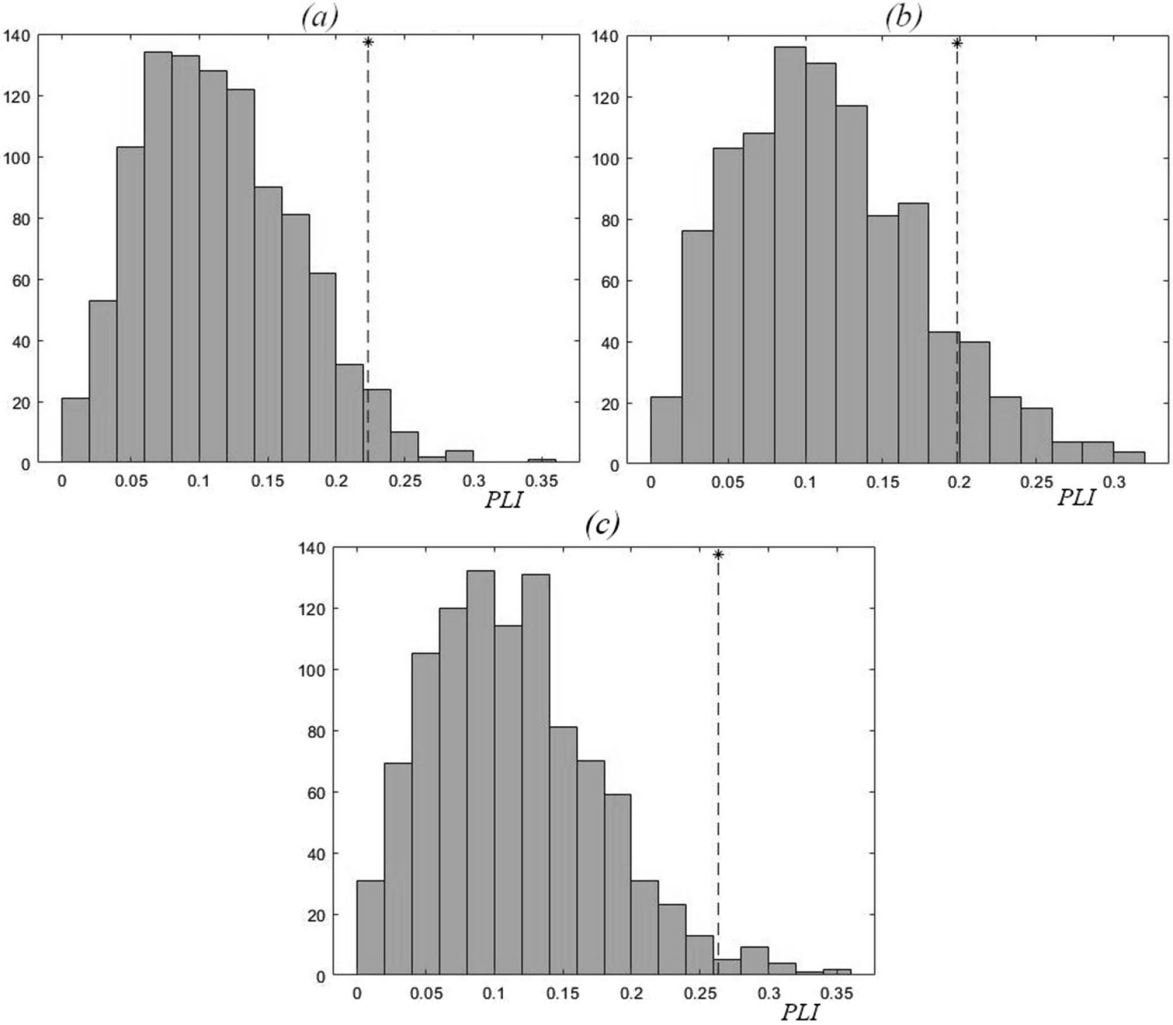
Values of PLI (shown by *) for 1993–2018, and the corresponding distributions of *PLI* values for the surrogate data resulted from 1000 random shuffles of the initial Chl and TP time series for each of the Naroch Lakes: (**a**) Lake Batorino (*PLI* = 0.22; *k* = 96%), (**b**) Lake Myastro (*PLI* = 0.20; *k* = 86%), (**c**) Lake Naroch (*PLI* = 0.26; *k* = 98%). Here the *t*_*n*_ moments correspond to the maximum values of these time series.

**Figure 5:**
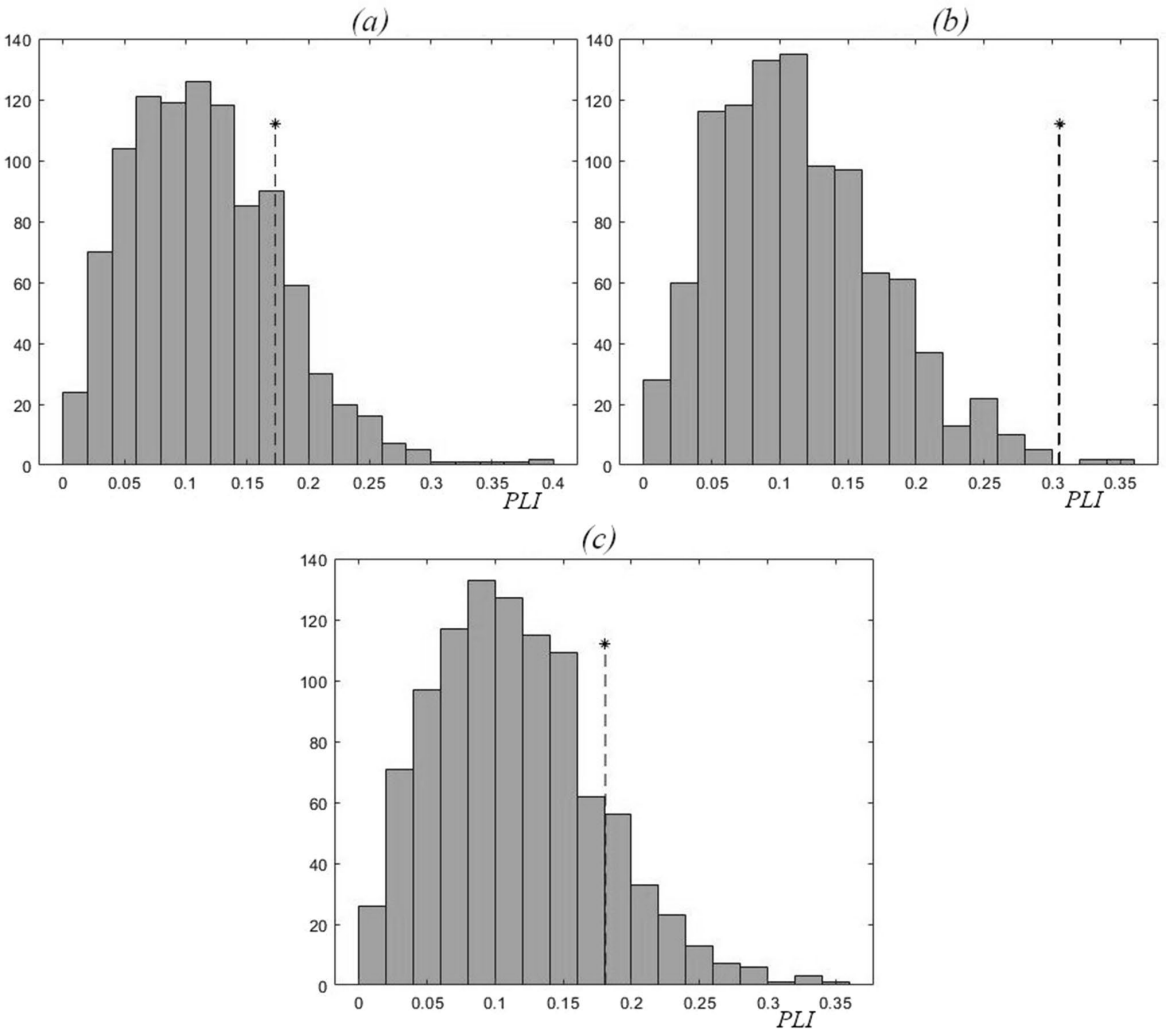
Values of *PLI* (shown by *) for 1993–2018, and the corresponding distributions of *PLI* values for the surrogate data resulted from 1000 random shuffles of the initial Chl and TP time series for each of the Naroch Lakes: (**a**) Lake Batorino (*PLI* = 0.17; *k* = 77%), (**b**) Lake Myastro (*PLI* = 0.30; *k* = 99%), (**c**) Lake Naroch (*PLI* = 0.18; *k* = 86%). Here the *t*_*n*_ moments correspond to the minimum values of these time series.

The results of the analysis of the phase relations between the Chl and TP time series for the values of *t*_*n*_ (period II 1993–2018), which are associated with the maximum values of these time series (Fig. 4) and with the minimum values (Fig. 5), suggest that the oscillations of Chl and TP concentrations in every lake of the Naroch system are actually synchronized in phase. Note that although the *PLI* values (Fig. 4) lie within the surrogate data distributions, nevertheless, the values of the corresponding significance values (*k* = 96% for Lake Batorino and *k* = 98% for Lake Naroch) indicate phase synchronization of the dynamics of Chl and TP concentrations in these water bodies. Note that *k* = 100% corresponds to such numerical values of *PLI* that are outside the distribution of surrogate data. However, in Lake Myastro (provided that the moments *t*_*n*_ correspond to the maxima of the Chl and TP time series) there is no such synchronization (since *k* = 86% < 95%). Nonetheless, the moments *t*_*n*_ correspond to the minima of the Chl and TP time series in Lake Myastro are synchronized in phase (PLI = 0.30; k = 99% see Fig. 5).

The results of the analysis of the phase relations between the Chl and TP time series for the values of *t*_*n*_, which are associated with the minimum values of these time series (see Fig. 5), suggest that the oscillations of Chl and TP concentrations in Lake Myastro, which turned out to be out of sync in Fig. 4, are actually synchronized in phase.

In general, the results of the analysis of the phase synchronization of the time series of Chl concentrations and the time series of TP concentrations, taking into account the values of *t*_*n*_, which correspond to both the minimum and maximum values of the concentrations of Chl and TP, allow us to conclude that these time series are phase synchronized in 1993–2018.

## Discussion

Many studies have been devoted to the problem of the occurrence of oscillations in ecological systems (see, for example^36,37^. In particular, Chl and TP oscillations can be driven by a plethora of factors, such as trophic interactions, input from the catchment area, temperature fluctuations. The subject of this paper, however, is not the process of variations in the concentrations of Chl and TP over time, but the change in the synchrony of their oscillations following the biological invasion and reduction of the phosphorus load.

The time series that we present here (Fig. 2) clearly show that the environmental measures that were carried out in the catchment area of the Naroch Lakes, as well as the invasion of zebra mussel *Dreissena polymorpha* Pallas, were followed by significant changes in the nature of Chl and TP oscillations. These significant changes, however, were not evident when examining the correlation between oscillations in Chl and TP concentrations. Indeed, such correlations took place in Lake Batorino and Lake Myastro both before the invasion of mussels and environmental measures (period I) and after (period II). At the same time, in Lake Naroch, the correlation between oscillations in Chl and TP concentrations was practically absent both during period I and during period II (Table 2).

In search of such a numerical parameter that would be able to reflect the changes characterizing the transition from period I to period II (Fig. 2), we turned to the estimation of the phase synchronization^24^ (but see also^38^) of Chl and TP oscillations. Note that, unlike the calculation of the correlation coefficient, the calculation of phase synchronization does not use the amplitude of oscillations and is indifferent to the trend. We used numerical estimates of the phase-locking index, *PLI* (see Methods), in order to find out the effect of changes in nutrient load on the phase synchronization of irregular oscillations (Fig. 2) of Chl and TP, concentrations in the Naroch Lakes. This analysis approach identified that during period I of high nutrient load (1979—1988), the oscillations of Chl and TP concentrations were not synchronized in phase (Fig. 3). Following a significant decrease in the nutrient load in 1993–2018 and the invasion of zebra mussel *Dreissena polymorpha*, there was a noticeable phase synchronization of Chl and TP oscillations (Figs. 4 and 5). It is important to note that when determining the level of phase synchronization, the *PLI* value must necessarily be compared with an array of surrogate data.

While TP as a limiting nutrient is known to impact Chl concentrations, there are also other factors that could be affecting the oscillations of Chl, such as temperature, exposure to sunlight, and trophic interactions. The occurrence of the Chl-TP synchronization during period II may be due to redistribution of the relative contribution of these factors, aggravated by influence of the invasion of zebra mussel Dreissena polymorpha. When zebra mussels invade, the plankton biomass decreases while the abundance of benthophage fish increases, and primary production is more affected by higher trophic levels than in ecosystems without zebra mussels^31,39^. Zebra mussel can become a determining factor in the phosphorus availability^40^ and thereby contribute to a greater dependence of phytoplankton development on phosphorus and reduce the influence of other factors. It was also shown that the transformation of the ecosystem of the Naroch Lakes has also affected the structure of phytoplankton communities. In particular, in Lake Naroch and Lake Myastro, the biomass of diatoms decreased during this transformation^41^. In this context, it is worth noting that the phase-locking index, *PLI*, that characterizes the phase synchronization of the processes under study, reflects the impact of the plethora of biological and hydrological factors on the relationship between the Chl dynamics and TP variations, and therefore can be considered as a holistic parameter.

Along with phosphorus, water temperature has a significant effect on the vital activity of phytoplankton^9,42,43,45^. In this regard, we did a comparative assessment of the relationship using *PLI*s between temperature and Chl oscillations in the Naroch Lake ecosystem before and after the transformation.

Oscillations of Chl and variations in the temperature averaged over the water column remained correlated with each other in both period I and period II despite the drastic changes that the ecosystem of the Naroch Lakes has undergone (Table 3). Additionally, the Naroch Lakes are highly susceptible to wind mixing, thus are considered polimictic (Table 1) water bodies. In these water bodies, there were significant correlations between the average temperature and the temperature oscillations at different depths^26^.

**Table 3:** The Spearman correlation coefficients between Chl a and mean temperature of the water column oscillations; *p* < 0.05 (*), *p* < 0.01 (**), *p* < 0.001 (***).

Water body	1979–1988	1993–2018
Lake Batorino	0.34* (n = 43)	0.24** (n = 120)
Lake Myastro	0.59*** (n = 45)	0.54*** (n = 126)
Lake Naroch	0.56*** (n = 45)	0.64*** (n = 127)

We took into account that Chl may exhibit a delayed response to environmental factors^44^. Here the time-lag between Chl a and temperature oscillations is equal to one time step, i.e. to one month.

The results of estimates of the *PLI* values in period I and period II are shown in Table 4. It is noteworthy that, unlike Chl and TP oscillations, synchronization between which was absent during period I (Fig. 3) and was manifested as statistically significant *PLI* values only during period II (Figs. 4 and 5), the Chl and water temperature oscillations demonstrated phase synchronization in both period I and period II (Table 4), and thus their synchronization could not be used as an indicator of the transformation in the ecosystem of the Naroch Lakes. It remains in this study that the *PLI* characterizing the synchronization of Chl and TP oscillations is a key indicator of the lake transformation. However, the mechanisms behind their synchrony require further study.

**Table 4:** *PLI* and the corresponding significance values *k*, which characterize the phase synchronization between temperature oscillations and oscillations in the chlorophyll a concentration in each of the Naroch Lakes.

Water body	*PLI*/*k* (period I)	*PLI*/*k* (period II)
Lake Batorino	0.4/98%	0.25/97%
Lake Myastro	0.46/98%	0.25/96%
Lake Naroch	0.33/99%	0.5/100%

Up to now, the phase synchronization of ecological processes remains an insufficiently investigated phenomenon. In this context, it is time to open the gates for a more comprehensive approach to the study of phase synchronization of ecological processes, which involves the use of both field monitoring data analysis and mathematical modeling methods to cover the relationships (including synchronization) between processes that are overlooked in environmental studies.

## Methods

### Field sampling

Water samples were collected monthly at specific monitoring points of the pelagic zones of the Naroch Lakes during the vegetative season (from May to October) using two-liter Ruttner sampler. The samples were collected from The samples were collected from six different depths (0.5, 3, 6, 8, 12 and 16 m) in Lake Naroch (54° 53′ 10.56" N, 26° 43′ 12.12" E), from four depths (0.5, 4, 7 and 9 m) in Lake Myastro (54° 52′ 0.91" N, 26° 52′ 49.86" E), and from three depths (0.5, 3 and 5 m) in Lake Batorino (54° 50′ 47.94" N, 26° 58′ 3.36" E). The water samples from all depths were mixed. Volumes of samples collected from different depths were proportional to the total estimated volume of the water in this horizon in the lake (according to bathymetry tests). Temperature was measured at all tested depths using a mercury deep-water thermometer with a scale resolution of 0.1 °C.

### Water chemistry

The suspended matter for determination of chlorophyll content (without correction for the presence of pheopigments) was collected on the nuclear membrane filters with a pore diameter of 1.5 μm. The analysis of chlorophyll was carried out by standard spectrophotometry. Pigments were extracted using 90% acetone. The chlorophyll concentration was calculated as described in^14,46^.

The concentration of the total phosphorus was determined after mineralization of a raw water sample with potassium persulfate in acid medium on water bath^47,48^.

### Correlation analysis

Spearman's Rank Correlation Coefficient was used as nonparametric measure of correlation strength. The Spearman correlation between two variables is equal to the Pearson correlation between the rank values of those two variables:

$\rho =\frac{cov(M,S)}{\sigma (M)\sigma (S)}$, where $cov(M,S)$ is the covariance of the rank variables, $\sigma (M)$ and $\sigma (S)$ are the standard deviations of the rank variables.

The significance was tested using t-test: $t=\rho \sqrt{\frac{n-2}{1-{\rho }^{2}}}$ with n − 2 degrees of freedom under the null hypothesis.

### The analysis of phase relations between time series

For irregular oscillations typical of the Chl and TP dynamics (Fig. 2) the phase of oscillations can be defined as the function^23^.

$$\varphi \left(t\right)=2\pi \left(n+\frac{t-{t}_{n}}{{t}_{n+1}-{t}_{n}}\right), {t}_{n}\le t<{t}_{n+1},$$

where *t* is time, *t*_*k*_ (*k* = *n*, *n* + 1) is the *k*-th point in time at which the oscillation under study reaches its maximum/minimum.

To assess the degree of synchronization of two oscillatory processes, a measure of synchronization, the phase-locking index (*PLI*) was suggested^24^. It is defined as

$$PLI=\frac{1}{N}\sum_{j=0}^{N-1}{e}^{i\Delta \varphi (j)},$$

where *N* is the number of measurements, and Δ*φ* is the phase difference between oscillatory processes. *PLI* is restricted to the interval [0, 1] and reaches 1 iff the time series are strictly synchronized whereas for unsynchronized time series (*i*.*e*. for a uniform distribution of Δ*φ*) *PLI* = 0.

In real data neither of these extreme values can be observed, but values between 0 and 1 are typical. Statistical significance testing must be done to establish whether a *PLI* value resulted from the analysis of phase relation between time series indicates a real dynamical coupling between the processes under study. Testing with surrogate data^35^ allows estimating how much synchronized the processes are.

The studied time series contained an insignificant amount of missing data (see Supporting Materials S1). The missing values were imputed by seasonally splitted missing value imputation (with interpolation) using the impute TS package^49,50^. Imputation algorithm splits the times series into seasons and afterwards performs imputation separately for each of the resulting time series datasets (each containing the data for one specific season). The time series obtained as a result of the imputation are shown in Fig. 2.

## Supplementary Information

Supplementary Tables.

## Data Availability

All data generated or analysed during this study are included in this published article [and its supplementary information files].
