# Supplementary material for: Phase synchronization of chlorophyll and total phosphorus oscillations as an indicator of the transformation of a lake ecosystem
Source: Sci Rep. 2022 Jul 13;12:11979. doi: 10.1038/s41598-022-16111-8 (PMC9279375; doi:10.1038/s41598-022-16111-8)
Supplement: Supplementary file 1 — Supplementary Tables. [file 41598_2022_16111_MOESM1_ESM.docx]

**Supplementary material**

Table SI1. Total phosphorus (mg/l)

|  | Lake Naroch | Lake Myastro | Lake Batorino |
| --- | --- | --- | --- |
| V 1979 | 0.0195 | 0.036 | 0.034 |
| VI 1979 | 0.0197 | 0.024 | 0.104 |
| VII 1979 | 0.0317 | 0.039 | 0.136 |
| VIII 1979 | 0.012 | 0.044 | 0.088 |
| IX 1979 | 0.0285 | 0.05 | 0.086 |
| X 1979 | 0.016 | 0.041 | 0.052 |
| V 1980 |  |  |  |
| VI 1980 | 0.04 | 0.06 | 0.079 |
| VII 1980 | 0.03 | 0.0895 | 0.09 |
| VIII 1980 | 0.0387 | 0.063 |  |
| IX 1980 |  |  |  |
| X 1980 | 0.035 | 0.066 | 0.071 |
| V 1981 | 0.0447 | 0.064 | 0.083 |
| VI 1981 | 0.013 | 0.055 | 0.055 |
| VII 1981 | 0.042 | 0.088 | 0.091 |
| VIII 1981 | 0.029 | 0.075 | 0.083 |
| IX 1981 | 0.04 | 0.042 | 0.092 |
| X 1981 | 0.033 | 0.064 | 0.071 |
| V 1982 | 0.021 | 0.065 | 0.108 |
| VI 1982 | 0.038 | 0.058 | 0.059 |
| VII 1982 | 0.024 | 0.041 | 0.079 |
| VIII 1982 | 0.035 | 0.117 | 0.1 |
| IX 1982 | 0.033 | 0.08 | 0.083 |
| X 1982 | 0.036 | 0.063 | 0.099 |
| V 1983 | 0.029 | 0.038 | 0.076 |
| VI 1983 | 0.023 | 0.04 | 0.043 |
| VII 1983 | 0.069 | 0.059 | 0.106 |
| VIII 1983 | 0.038 | 0.081 | 0.188 |
| IX 1983 | 0.033 | 0.109 | 0.124 |
| X 1983 | 0.029 |  |  |
| V 1984 | 0.025 | 0.039 | 0.069 |
| VI 1984 | 0.03 | 0.044 | 0.103 |
| VII 1984 | 0.015 | 0.049 | 0.078 |
| VIII 1984 | 0.033 | 0.064 | 0.079 |
| IX 1984 | 0.026 | 0.065 | 0.101 |
| X 1984 | 0.023 | 0.053 | 0.104 |
| V 1985 | 0.03 | 0.056 | 0.083 |
| VI 1985 | 0.087 | 0.053 | 0.169 |
| VII 1985 | 0.027 | 0.043 | 0.12 |
| VIII 1985 | 0.023 | 0.054 | 0.105 |
| IX 1985 | 0.02 | 0.042 | 0.092 |
| X 1985 | 0.023 | 0.046 | 0.062 |
| V 1986 | 0.013 | 0.026 | 0.019 |
| VI 1986 | 0.015 | 0.036 | 0.071 |
| VII 1986 | 0.021 | 0.035 | 0.076 |
| VIII 1986 | 0.011 | 0.0505 | 0.069 |
| IX 1986 |  | 0.042 |  |
| X 1986 | 0.015 | 0.031 | 0.046 |
| V 1987 |  |  |  |
| VI 1987 | 0.01 | 0.035 | 0.095 |
| VII 1987 | 0.016 | 0.049 | 0.08 |
| VIII 1987 | 0.02 | 0.049 | 0.084 |
| IX 1987 | 0.025 | 0.04 | 0.066 |
| X 1987 | 0.016 | 0.034 | 0.053 |
| V 1988 | 0.011 | 0.029 | 0.057 |
| VI 1988 | 0.014 | 0.032 | 0.068 |
| VII 1988 | 0.017 | 0.032 | 0.053 |
| VIII 1988 | 0.011 | 0.042 | 0.067 |
| IX 1988 | 0.025 | 0.046 | 0.073 |
| X 1988 | 0.021 | 0.041 | 0.06 |
|  |  |  |  |
| V 1993 | 0.015 | 0.031 | 0.05 |
| VI 1993 | 0.021 | 0.024 | 0.0475 |
| VII 1993 | 0.018 | 0.035 | 0.037 |
| VIII 1993 | 0.02 | 0.049 | 0.045 |
| IX 1993 | 0.012 | 0.039 | 0.03 |
| X 1993 | 0.012 | 0.04 | 0.028 |
| V 1994 | 0.014 | 0.03 | 0.063 |
| VI 1994 | 0.014 | 0.03 | 0.065 |
| VII 1994 | 0.016 | 0.03 | 0.035 |
| VIII 1994 | 0.02 | 0.059 | 0.053 |
| IX 1994 | 0.013 | 0.042 | 0.031 |
| X 1994 | 0.014 | 0.045 | 0.032 |
| V 1995 | 0.016 | 0.022 | 0.043 |
| VI 1995 | 0.012 | 0.02 | 0.044 |
| VII 1995 | 0.013 | 0.028 | 0.045 |
| VIII 1995 | 0.017 | 0.051 | 0.047 |
| IX 1995 | 0.016 | 0.056 | 0.052 |
| X 1995 | 0.015 | 0.036 | 0.029 |
| V 1996 | 0.019 | 0.027 | 0.032 |
| VI 1996 | 0.017 | 0.027 | 0.037 |
| VII 1996 | 0.011 | 0.028 | 0.053 |
| VIII 1996 | 0.014 | 0.039 | 0.046 |
| IX 1996 | 0.011 | 0.047 | 0.036 |
| X 1996 | 0.012 | 0.031 | 0.028 |
| V 1997 | 0.02 | 0.029 | 0.042 |
| VI 1997 | 0.022 | 0.036 | 0.053 |
| VII 1997 | 0.017 | 0.0375 | 0.0485 |
| VIII 1997 | 0.018 | 0.035 | 0.038 |
| IX 1997 | 0.016 | 0.039 | 0.04 |
| X 1997 | 0.012 | 0.03 | 0.031 |
| V 1998 | 0.011 | 0.021 | 0.037 |
| VI 1998 | 0.014 | 0.026 | 0.036 |
| VII 1998 | 0.014 | 0.034 | 0.038 |
| VIII 1998 | 0.018 | 0.046 | 0.04 |
| IX 1998 | 0.019 | 0.039 | 0.04 |
| X 1998 | 0.011 | 0.026 | 0.023 |
| V 1999 | 0.022 | 0.03 | 0.052 |
| VI 1999 | 0.018 | 0.03 | 0.037 |
| VII 1999 | 0.015 | 0.051 | 0.042 |
| VIII 1999 | 0.017 | 0.046 | 0.053 |
| IX 1999 | 0.021 | 0.037 | 0.068 |
| X 1999 | 0.015 |  |  |
| V 2000 | 0.015 | 0.027 | 0.036 |
| VI 2000 | 0.014 | 0.02 | 0.04 |
| VII 2000 | 0.009 | 0.03 | 0.039 |
| VIII 2000 | 0.014 | 0.035 | 0.04 |
| IX 2000 | 0.016 | 0.034 | 0.033 |
| X 2000 | 0.014 | 0.031 | 0.028 |
| V 2001 | 0.011 | 0.024 | 0.03 |
| VI 2001 | 0.011 | 0.026 | 0.039 |
| VII 2001 | 0.017 | 0.03 | 0.039 |
| VIII 2001 | 0.021 | 0.051 | 0.043 |
| IX 2001 | 0.009 | 0.041 | 0.031 |
| X 2001 |  |  |  |
| V 2002 | 0.01 | 0.02 | 0.034 |
| VI 2002 | 0.011 | 0.014 | 0.037 |
| VII 2002 | 0.018 | 0.018 | 0.041 |
| VIII 2002 | 0.018 | 0.038 | 0.045 |
| IX 2002 | 0.018 | 0.064 | 0.04 |
| X 2002 | 0.016 | 0.04 | 0.0325 |
| V 2003 | 0.014 | 0.023 | 0.024 |
| VI 2003 | 0.013 | 0.025 | 0.044 |
| VII 2003 | 0.016 | 0.027 | 0.025 |
| VIII 2003 | 0.018 | 0.041 | 0.03 |
| IX 2003 | 0.012 | 0.028 | 0.022 |
| X 2003 | 0.015 | 0.032 | 0.033 |
| V 2004 | 0.011 | 0.02 | 0.032 |
| VI 2004 | 0.011 | 0.027 | 0.039 |
| VII 2004 | 0.01 | 0.026 | 0.032 |
| VIII 2004 | 0.018 | 0.036 | 0.036 |
| IX 2004 | 0.011 | 0.034 | 0.039 |
| X 2004 |  |  |  |
| V 2005 | 0.0075 | 0.017 | 0.026 |
| VI 2005 | 0.012 | 0.03 | 0.039 |
| VII 2005 | 0.015 | 0.036 | 0.034 |
| VIII 2005 | 0.028 | 0.047 | 0.031 |
| IX 2005 | 0.017 | 0.053 | 0.03 |
| X 2005 | 0.01 | 0.029 | 0.0280 |
| V 2006 | 0.0105 | 0.022 | 0.031 |
| VI 2006 | 0.012 | 0.021 | 0.033 |
| VII 2006 | 0.012 | 0.047 | 0.035 |
| VIII 2006 | 0.016 | 0.038 | 0.029 |
| IX 2006 | 0.014 | 0.045 | 0.026 |
| X 2006 | 0.012 | 0.042 | 0.022 |
| V 2007 | 0.013 | 0.019 | 0.029 |
| VI 2007 | 0.013 | 0.027 | 0.033 |
| VII 2007 | 0.014 | 0.024 | 0.025 |
| VIII 2007 | 0.017 | 0.029 | 0.034 |
| IX 2007 | 0.013 | 0.038 | 0.034 |
| X 2007 | 0.0140 | 0.036 | 0.036 |
| V 2008 | 0.019 | 0.046 | 0.055 |
| VI 2008 | 0.009 | 0.022 | 0.043 |
| VII 2008 | 0.018 | 0.041 | 0.038 |
| VIII 2008 | 0.018 | 0.057 | 0.035 |
| IX 2008 | 0.023 | 0.055 | 0.028 |
| X 2008 | 0.016 | 0.039 | 0.034 |
| V 2009 | 0.0105 | 0.023 | 0.025 |
| VI 2009 | 0.012 | 0.027 | 0.035 |
| VII 2009 | 0.019 | 0.05 | 0.037 |
| VIII 2009 | 0.015 | 0.087 | 0.048 |
| IX 2009 | 0.012 | 0.047 | 0.028 |
| X 2009 |  | 0.055 | 0.029 |
| V 2010 | 0.019 | 0.028 | 0.035 |
| VI 2010 | 0.012 | 0.027 | 0.035 |
| VII 2010 | 0.017 | 0.041 | 0.029 |
| VIII 2010 | 0.015 | 0.127 | 0.025 |
| IX 2010 | 0.02 | 0.069 | 0.027 |
| X 2010 | 0.012 | 0.039 | 0.024 |
| V 2011 | 0.01 | 0.026 | 0.02 |
| VI 2011 | 0.01 | 0.024 | 0.034 |
| VII 2011 | 0.015 | 0.026 | 0.029 |
| VIII 2011 | 0.015 | 0.054 | 0.0340 |
| IX 2011 | 0.013 | 0.038 | 0.029 |
| X 2011 | 0.009 | 0.026 | 0.029 |
| V 2012 | 0.011 | 0.018 | 0.027 |
| VI 2012 | 0.008 | 0.022 | 0.021 |
| VII 2012 | 0.01 | 0.017 | 0.04 |
| VIII 2012 | 0.02 | 0.034 | 0.028 |
| IX 2012 | 0.01 | 0.033 | 0.04 |
| X 2012 | 0.01 | 0.037 | 0.044 |
| V 2013 | 0.015 | 0.018 | 0.026 |
| VI 2013 | 0.01 | 0.013 | 0.026 |
| VII 2013 | 0.007 | 0.021 | 0.023 |
| VIII 2013 | 0.011 | 0.027 | 0.011 |
| IX 2013 | 0.018 | 0.032 | 0.031 |
| X 2013 | 0.011 | 0.027 | 0.022 |
| V 2014 | 0.01 | 0.012 | 0.023 |
| VI 2014 | 0.013 | 0.023 | 0.032 |
| VII 2014 | 0.011 | 0.023 | 0.03 |
| VIII 2014 | 0.012 | 0.05 | 0.031 |
| IX 2014 | 0.015 | 0.025 | 0.035 |
| X 2014 | 0.014 | 0.026 | 0.038 |
| V 2015 | 0.01 | 0.024 | 0.037 |
| VI 2015 | 0.01 | 0.026 | 0.036 |
| VII 2015 | 0.013 | 0.032 | 0.037 |
| VIII 2015 | 0.017 | 0.045 | 0.035 |
| IX 2015 | 0.012 | 0.038 | 0.038 |
| X 2015 | 0.01 | 0.026 | 0.032 |
| V 2016 |  | 0.022 | 0.035 |
| VI 2016 | 0.009 | 0.021 | 0.039 |
| VII 2016 | 0.015 | 0.035 | 0.036 |
| VIII 2016 | 0.015 | 0.035 | 0.036 |
| IX 2016 | 0.013 | 0.044 | 0.046 |
| X 2016 | 0.015 | 0.039 | 0.054 |
| V 2017 | 0.009 | 0.021 | 0.023 |
| VI 2017 | 0.01 | 0.023 | 0.035 |
| VII 2017 | 0.012 | 0.024 | 0.02 |
| VIII 2017 | 0.008 | 0.026 | 0.034 |
| IX 2017 | 0.007 | 0.02 | 0.025 |
| X 2017 | 0.008 | 0.021 | 0.031 |
| V 2018 | 0.02 | 0.02 | 0.03 |
| VI 2018 | 0.01 | 0.02 | 0.04 |
| VII 2018 | 0.01 | 0.02 | 0.03 |
| VIII 2018 | 0.01 | 0.07 | 0.05 |
| IX 2018 |  | 0.05 | 0.04 |
| X 2018 | 0.01 | 0.02 | 0.02 |

Table SI2. Chlorophyll a (mkg/l)

|  | Lake Naroch | Lake Myastro | Lake Batorino |
| --- | --- | --- | --- |
| V 1979 | 6.6833 | 16.1 | 54.22 |
| VI 1979 | 2.165 | 13.4 | 45.86 |
| VII 1979 | 3.2433 | 15.2 | 73.1 |
| VIII 1979 | 4.275 | 30 | 93.57 |
| IX 1979 | 7.11 | 28.78 | 98.48 |
| X 1979 | 5.79 | 21.8 | 36.3 |
| V 1980 | 7.3 | 21.39 | 35.02 |
| VI 1980 | 1.35 | 7.91 |  |
| VII 1980 | 1.89 | 28.98 | 55.22 |
| VIII 1980 | 4.9 |  | 81.14 |
| IX 1980 |  |  |  |
| X 1980 | 12.8 | 19.68 | 119.68 |
| V 1981 | 2.68 | 14.23 | 41.6 |
| VI 1981 | 1.54 | 13.57 | 27.6 |
| VII 1981 | 1.61 | 5.3 | 33.75 |
| VIII 1981 | 1.78 | 23.35 | 58.43 |
| IX 1981 |  | 60.72 | 75 |
| X 1981 | 5.78 | 23.32 | 34.63 |
| V 1982 | 3.94 | 12.5 | 12.54 |
| VI 1982 | 1.22 | 14.6 | 17.95 |
| VII 1982 | 1.58 | 10.9 | 23.57 |
| VIII 1982 | 4.66 | 25.6 | 47.19 |
| IX 1982 | 3.69 | 23.5 | 57.78 |
| X 1982 | 6.01 | 25.2 | 26.55 |
| V 1983 | 2.5 | 13.07 | 14.96 |
| VI 1983 | 3.66 | 17.57 | 22.31 |
| VII 1983 | 2.89 | 13.8 | 82.96 |
| VIII 1983 | 5.69 | 38.13 | 116.4 |
| IX 1983 | 10.57 | 63.33 | 164.1 |
| X 1983 | 9.29 | 28.46 | 74.6 |
| V 1984 | 8.06 | 19.4 | 35.97 |
| VI 1984 | 2.82 | 14.4 | 115.3 |
| VII 1984 | 1.66 | 19.1 | 42.23 |
| VIII 1984 | 5.69 | 29.9 | 58.1 |
| IX 1984 | 9.95 | 34.63 | 70.64 |
| X 1984 | 7.14 | 18.05 | 51.86 |
| V 1985 | 5.88 | 12.37 | 29.73 |
| VI 1985 | 3.07 | 8.41 | 78.89 |
| VII 1985 | 4.32 | 12.6 | 53.28 |
| VIII 1985 | 4.2 | 18.63 | 60.98 |
| IX 1985 | 9.11 | 24.2 | 58.26 |
| X 1985 | 9.09 | 17.87 | 26.79 |
| V 1986 | 3.61 | 17.04 | 12.45 |
| VI 1986 | 1.37 | 9.72 | 27.03 |
| VII 1986 | 4.52 | 14.13 | 30.75 |
| VIII 1986 | 3.61 | 13.94 | 59.91 |
| IX 1986 |  | 24.4 |  |
| X 1986 | 2.23 | 7.4 | 15.56 |
| V 1987 | 4.99 | 15.09 | 24.83 |
| VI 1987 | 3.44 | 15.4 | 45.54 |
| VII 1987 |  |  |  |
| VIII 1987 | 3.93 | 29.91 | 36.4 |
| IX 1987 | 5.71 | 14.72 | 32.57 |
| X 1987 | 4.63 | 12.81 | 27.44 |
| V 1988 | 12.1 | 15.79 | 18.68 |
| VI 1988 | 1.7 | 5.47 | 26.72 |
| VII 1988 | 1.91 | 3.89 | 18.29 |
| VIII 1988 | 3.89 | 23.71 | 36.72 |
| IX 1988 | 5.98 | 14.73 | 29.5 |
| X 1988 | 5.62 | 21.56 | 22.1 |
|  |  |  |  |
| V 1993 | 0.84 | 3.07 | 15.96 |
| VI 1993 | 0.9 | 1.55 | 12.4 |
| VII 1993 | 0.57 | 4.78 | 5.17 |
| VIII 1993 | 1.23 | 3.52 | 8.96 |
| IX 1993 | 2.01 | 2.65 | 9.64 |
| X 1993 | 0.95 | 1.81 | 2.76 |
| V 1994 | 0.86 | 0.53 | 13.27 |
| VI 1994 | 0.61 | 0.94 | 16.64 |
| VII 1994 | 0.66 | 3.97 | 7.26 |
| VIII 1994 | 1.62 | 3.64 | 25.4 |
| IX 1994 | 3.59 | 2.34 | 9.98 |
| X 1994 | 1.33 | 1.11 | 6.94 |
| V 1995 | 1.26 | 1.03 | 6.07 |
| VI 1995 | 0.73 | 1.51 | 11.45 |
| VII 1995 | 1.1 | 17.4 | 11.26 |
| VIII 1995 | 1.1 | 3.48 | 12.13 |
| IX 1995 | 1.530 | 3.51 | 13.51 |
| X 1995 | 1.6 | 2.87 | 13.25 |
| V 1996 | 1.46 | 2.53 | 7.18 |
| VI 1996 | 1.22 | 1.68 | 7.71 |
| VII 1996 | 0.78 | 6.68 | 14.49 |
| VIII 1996 | 1.24 | 5.59 | 9.12 |
| IX 1996 | 1.48 | 2.6 | 16.7 |
| X 1996 | 1.1 | 4.04 | 5.52 |
| V 1997 | 0.9 | 2.1 | 4.7 |
| VI 1997 | 0.3 | 0.73 | 4.06 |
| VII 1997 | 0.73 | 4.59 | 7.43 |
| VIII 1997 | 1.03 | 5.76 | 7.58 |
| IX 1997 | 1.36 | 5.63 | 9 |
| X 1997 | 1.43 | 3.33 | 6.6 |
| V 1998 | 1.03 | 2.69 | 5.68 |
| VI 1998 | 1.22 | 2.32 | 12.96 |
| VII 1998 | 1.49 | 7.07 | 9.5 |
| VIII 1998 | 2.26 | 7.88 | 9.8 |
| IX 1998 | 1.57 | 6.11 | 11.15 |
| X 1998 | 1.65 | 2.55 | 6.45 |
| V 1999 | 1.08 | 4.5 | 4.18 |
| VI 1999 | 1.02 | 1.12 | 5.05 |
| VII 1999 | 0.82 | 5 | 9.08 |
| VIII 1999 | 1.68 | 9.16 | 9.16 |
| IX 1999 | 1.58 | 2.72 | 8.5 |
| X 1999 | 1.76 |  |  |
| V 2000 | 1.05 | 2.81 | 7.45 |
| VI 2000 | 0.66 | 0.47 | 5.72 |
| VII 2000 | 0.85 | 1.83 | 2.95 |
| VIII 2000 | 1.37 | 2.28 | 4.68 |
| IX 2000 | 0.89 | 2.65 | 4.39 |
| X 2000 | 0.98 | 2.8 | 2.89 |
| V 2001 | 0.83 | 2.5 | 3.81 |
| VI 2001 | 0.36 | 0.42 | 6.61 |
| VII 2001 | 0.8 | 3.85 | 8.28 |
| VIII 2001 | 0.89 | 13.47 | 15.17 |
| IX 2001 | 1.45 | 7.47 | 8.73 |
| X 2001 |  |  |  |
| V 2002 | 0.9 | 5.6 | 13.85 |
| VI 2002 | 1.6 | 2.47 | 14.23 |
| VII 2002 | 1.51 | 4.09 | 14.38 |
| VIII 2002 | 2.48 | 4.98 | 21.04 |
| IX 2002 | 2.02 | 5.63 | 10.76 |
| X 2002 | 3.19 | 1.65 | 14.5 |
| V 2003 | 1.42 | 1.39 | 6.41 |
| VI 2003 | 0.81 | 2.02 | 8.16 |
| VII 2003 | 1.79 | 3.99 | 10.25 |
| VIII 2003 | 1.71 | 7.17 | 15.84 |
| IX 2003 | 2.33 | 3.61 | 3.61 |
| X 2003 | 2.19 | 2.43 | 6.93 |
| V 2004 | 2.25 | 1.89 | 10.51 |
| VI 2004 | 1.52 | 9 | 8.06 |
| VII 2004 | 0.93 | 4.1 | 8.61 |
| VIII 2004 | 1.76 | 11.6 | 10.4 |
| IX 2004 | 1.58 | 6.79 | 10.21 |
| X 2004 |  |  |  |
| V 2005 | 2.02 | 2.69 | 12.59 |
| VI 2005 | 1.15 | 5.44 | 13.65 |
| VII 2005 | 1.21 | 5.77 | 11.98 |
| VIII 2005 | 3.99 | 13.26 | 18.28 |
| IX 2005 | 2.35 | 10.23 | 21.09 |
| X 2005 | 2.96 | 1.47 |  |
| V 2006 | 0.6667 |  |  |
| VI 2006 | 0.78 | 4.7 | 6.38 |
| VII 2006 | 1.0925 | 7 | 15.95 |
| VIII 2006 | 2.19 |  |  |
| IX 2006 | 2.16 | 5.53 | 5.67 |
| X 2006 | 1.32 | 4.45 | 4.95 |
| V 2007 | 1.12667 | 1.24 | 8.21 |
| VI 2007 | 0.475 | 1.52 | 5.85 |
| VII 2007 | 0.95 | 3.97 | 6.47 |
| VIII 2007 | 2.0875 | 5.4 | 20.18 |
| IX 2007 | 1.72 | 4.42 | 10.79 |
| X 2007 | 1.325 | 3.43 | 12.87 |
| V 2008 | 1.19 | 0.63 | 10.52 |
| VI 2008 | 0.86 | 1.82 | 10.43 |
| VII 2008 | 0.81 | 4.59 | 7.94 |
| VIII 2008 | 0.95 | 3.17 | 4.91 |
| IX 2008 | 1.71 | 3.23 | 8.83 |
| X 2008 | 1.13 | 2.61 | 8.41 |
| V 2009 | 0.63 | 1.29 | 7.96 |
| VI 2009 | 0.41 | 3.49 | 6.66 |
| VII 2009 | 0.99 | 3.53 |  |
| VIII 2009 | 1.82 | 10.18 | 10.77 |
| IX 2009 | 1.08 | 3.8 | 4.74 |
| X 2009 |  | 2.22 | 6.38 |
| V 2010 | 2.31 | 8.44 | 9.32 |
| VI 2010 | 0.82 | 0.71 | 11.47 |
| VII 2010 | 0.61 | 2.9 | 5.5 |
| VIII 2010 | 1.04 | 3.91 | 5.68 |
| IX 2010 | 1.35 | 21.87 | 7.43 |
| X 2010 | 2.12 | 3.4 | 12.99 |
| V 2011 | 1.29 | 1.85 | 6.4 |
| VI 2011 | 0.67 | 1.05 | 7.61 |
| VII 2011 | 1.1 | 2.31 | 10.54 |
| VIII 2011 | 1.7 | 5.82 | 9.88 |
| IX 2011 | 2.96 | 11.35 | 5.92 |
| X 2011 | 1.48 | 6.67 | 5.79 |
| V 2012 | 0.87 | 1.08 | 8.25 |
| VI 2012 | 0.435 | 2.51 | 8.25 |
| VII 2012 | 0.81 | 2.65 | 6 |
| VIII 2012 | 1.18 | 5.92 | 9.99 |
| IX 2012 | 1.87 | 5.19 | 9.22 |
| X 2012 | 1.4 | 15.32 | 10.3 |
| V 2013 | 1.24 | 5.22 | 4.88 |
| VI 2013 | 0.69 | 2.41 | 8.76 |
| VII 2013 | 0.59 | 2.53 | 8.2 |
| VIII 2013 | 1.59 | 4.36 | 10.82 |
| IX 2013 | 2.12 | 5.52 | 10.69 |
| X 2013 | 1.61 | 3.15 | 5.05 |
| V 2014 | 0.58 | 1.66 | 4.74 |
| VI 2014 | 0.66 | 6.5 | 5.14 |
| VII 2014 | 0.88 | 2.09 | 7.87 |
| VIII 2014 | 1.28 | 2.78 | 12.43 |
| IX 2014 | 1.35 | 2.25 | 8.09 |
| X 2014 | 1.17 | 2.65 | 5.17 |
| V 2015 | 0.73 | 4.96 | 4.75 |
| VI 2015 | 1.3 | 3.76 | 9.15 |
| VII 2015 | 0.98 | 5.75 | 8.84 |
| VIII 2015 | 1.69 | 9.73 | 10.95 |
| IX 2015 | 2 | 6.88 | 10.2 |
| X 2015 | 1.29 | 5.38 | 6.1 |
| V 2016 | 0.64 | 1.81 | 9.25 |
| VI 2016 | 0.63 | 2.33 | 9.95 |
| VII 2016 | 0.53 | 6.17 | 5.5 |
| VIII 2016 | 1.1 | 5.28 | 12.69 |
| IX 2016 | 1.9 | 5.43 | 18.58 |
| X 2016 | 1.79 | 2.49 | 17.76 |
| V 2017 | 1.44 | 4.56 | 5 |
| VI 2017 | 0.35 | 4.34 | 5.44 |
| VII 2017 | 0.59 | 4.56 | 5.97 |
| VIII 2017 | 2.15 | 6.9 | 8.76 |
| IX 2017 | 3.01 | 7.52 | 15.38 |
| X 2017 | 1.31 | 4.3 | 9.43 |
| V 2018 | 0.72 | 5.01 | 8.4 |
| VI 2018 | 0.59 | 4.68 | 8.82 |
| VII 2018 | 0.8 | 3.22 | 3.94 |
| VIII 2018 | 0.99 | 5.37 | 12.17 |
| IX 2018 | 1.52 | 9.77 | 5.63 |
| X 2018 | 1.93 | 9.87 | 7.22 |

Table SI3. Temperature (˚˚˚ C)

|  | Lake Naroch | Lake Myastro | Lake Batorino |
| --- | --- | --- | --- |
| V 1979 | 8.5056 | 8.2125 | 11.783 |
| VI 1979 | 15.378 | 16.25 | 20.967 |
| VII 1979 | 15.356 | 17.2625 | 17.5 |
| VIII 1979 | 17.6125 | 18.1625 | 18.067 |
| IX 1979 | 16.775 | 15.9 | 15.2 |
| X 1979 |  | 8.9 | 8.733 |
| V 1980 | 7.333 | 7.975 | 9.683 |
| VI 1980 | 15.83 | 17.5875 |  |
| VII 1980 | 16.875 | 17.325 | 16.783 |
| VIII 1980 | 17.7167 | 17.367 | 16.3 |
| IX 1980 | 14.10833 | 13.725 |  |
| X 1980 | 9.5 | 10.5 | 8.3667 |
| V 1981 | 9.7167 | 11.675 | 14.383 |
| VI 1981 | 14.4583 | 16.0625 | 15.667 |
| VII 1981 | 18.575 | 19.9125 | 19.55 |
| VIII 1981 | 19.483 | 19.95 | 20.1667 |
| IX 1981 | 12.883 | 11.825 | 13.1 |
| X 1981 | 10.5 | 10.95 | 9.9 |
| V 1982 | 9.15 | 11.2625 | 12.883 |
| VI 1982 | 13.4167 | 13.975 | 14.1667 |
| VII 1982 | 17.6 | 17.6875 | 19.1 |
| VIII 1982 | 18.29 | 18.6 | 17.267 |
| IX 1982 | 15.6 | 15.2125 | 14.683 |
| X 1982 | 11.175 | 10.65 | 8.7 |
| V 1983 | 11.125 | 13.525 | 17.13 |
| VI 1983 | 14.95 | 16.9125 | 17.217 |
| VII 1983 | 18.84167 | 19.9875 | 21.4 |
| VIII 1983 | 18.1 | 18.867 | 18.967 |
| IX 1983 | 16.15 | 15.6625 | 15.65 |
| X 1983 | 10.1 | 9.1 | 8.6 |
| V 1984 | 7.9583 | 10.0625 | 10.43 |
| VI 1984 | 12.783 | 14.9 | 14.1 |
| VII 1984 | 16.2167 | 17.6375 | 19.05 |
| VIII 1984 | 17.6917 | 18.2125 | 18.33 |
| IX 1984 | 13.7 |  | 12.05 |
| X 1984 | 10.1 | 8 | 5.7 |
| V 1985 | 9.45 | 11.55 | 13.433 |
| VI 1985 | 13.6 | 16.1 | 16.167 |
| VII 1985 | 16.8167 | 18.475 | 20.23 |
| VIII 1985 | 19.167 | 19.6 | 19.83 |
| IX 1985 | 15.1 | 13.8 | 9.3 |
| X 1985 | 10.85 | 10.025 | 9.4 |
| V 1986 | 7.6 | 10.625 | 15.167 |
| VI 1986 | 15.5167 | 18.0625 | 20.9 |
| VII 1986 | 16.83 | 16.875 | 16.93 |
| VIII 1986 |  | 17.975 | 19.9 |
| IX 1986 |  |  |  |
| X 1986 | 7.6 | 7.1 | 6.33 |
| V 1987 | 8.6 | 10.15 | 12.2 |
| VI 1987 | 15.083 | 17.6 | 19.43 |
| VII 1987 | 16.13 | 17.525 | 17.1 |
| VIII 1987 | 16.267 | 15.475 | 15.3 |
| IX 1987 | 14.2 | 13.2 | 12.7 |
| X 1987 | 8.4 |  | 8 |
| V 1988 | 10.73 | 11.825 | 14.43 |
| VI 1988 | 14.683 | 18.775 | 20.633 |
| VII 1988 | 17.8167 | 19.55 | 22.6 |
| VIII 1988 | 17.051 | 17.625 | 17.633 |
| IX 1988 | 15.368 | 15.025 | 14.3 |
| X 1988 | 10.836 | 10.3 | 9.3 |
|  |  |  |  |
| V 1993 | 11.3 | 13.05 | 18.1 |
| VI 1993 | 13.783 | 15.3 | 17.6 |
| VII 1993 | 17.3 | 17.925 | 18 |
| VIII 1993 | 18.55 | 18.65 | 19 |
| IX 1993 | 12.2 | 11.525 | 9.93 |
| X 1993 | 9.367 | 8.925 | 9.3 |
| V 1994 | 12.267 | 13.875 | 14.6 |
| VI 1994 | 14.53 | 15.825 | 16.4 |
| VII 1994 | 18.083 | 19.325 | 21.4 |
| VIII 1994 | 19.5 | 20.425 | 21.4 |
| IX 1994 | 15.6 | 15.275 | 14.7 |
| X 1994 | 6.6 | 7.7 | 5.967 |
| V 1995 | 8.8167 | 10.2 | 10.767 |
| VI 1995 | 17.067 | 17.325 | 21.3 |
| VII 1995 | 16.43 | 18.775 | 18.9 |
| VIII 1995 | 18.3 | 19.175 | 20.93 |
| IX 1995 | 17.25 | 17.2 | 16.3 |
| X 1995 | 11.5 | 10.9 | 10.6 |
| V 1996 | 9.63 | 12.975 | 17.43 |
| VI 1996 | 14.05 | 17.3 | 19.93 |
| VII 1996 | 17.3 | 18.275 | 19.13 |
| VIII 1996 | 19.3167 | 19.3 | 19 |
| IX 1996 | 16.3 | 15.5 | 13.4 |
| X 1996 | 9.85 | 9.225 | 8.4 |
| V 1997 | 8.9167 | 10.85 | 12.967 |
| VI 1997 | 13.825 | 15.95 | 17.83 |
| VII 1997 | 18.6583 | 19.65 | 20.43 |
| VIII 1997 | 20.167 | 21.4 | 22.43 |
| IX 1997 | 16.67 | 16.0125 | 14.8 |
| X 1997 | 11.7 | 8.7 | 8.7 |
| V 1998 | 10.03 | 12.9 | 15.167 |
| VI 1998 | 17.7167 | 18.45 | 22.4 |
| VII 1998 | 17.683 | 18.15 | 18.4 |
| VIII 1998 | 18.183 | 17.975 | 17.93 |
| IX 1998 | 14.8 | 14.5 | 13.867 |
| X 1998 | 7.8 | 7.2 | 6.9 |
| V 1999 | 9.35 | 10.55 | 11.2 |
| VI 1999 | 15.683 | 17.85 | 19.53 |
| VII 1999 | 18.89 | 21.467 | 23.43 |
| VIII 1999 | 19.089 | 19.65 | 19.63 |
| IX 1999 | 17.1 | 17.4 | 16.8 |
| X 1999 | 10.483 |  |  |
| V 2000 | 6.9167 | 11.175 | 14.1 |
| VI 2000 | 14.43 | 17.275 | 18.93 |
| VII 2000 | 16.63 | 18 | 21.03 |
| VIII 2000 | 18.4 | 18.925 | 19.6 |
| IX 2000 | 18.13 | 17.875 | 17.4 |
| X 2000 | 12.7167 | 12.05 | 11.3 |
| V 2001 | 8.45 | 11.075 | 14.567 |
| VI 2001 | 14.2167 | 16.35 | 17.8 |
| VII 2001 | 20.3 | 21.0375 | 23.583 |
| VIII 2001 | 19.03 | 20.55 | 20 |
| IX 2001 | 14 | 11 | 11.9 |
| X 2001 |  |  |  |
| V 2002 | 12.967 | 15.35 | 17.23 |
| VI 2002 | 16.983 | 17.875 | 19.43 |
| VII 2002 | 20.53 | 21.075 | 22.6 |
| VIII 2002 | 20.467 | 20.8 | 20.1 |
| IX 2002 | 17.1 | 13.3 | 11.4 |
| X 2002 | 6.75 | 5.975 | 4.67 |
| V 2003 | 8.767 | 11.725 | 14.3 |
| VI 2003 | 14.55 | 17.4 | 17.8 |
| VII 2003 | 18.3167 | 19.275 | 19 |
| VIII 2003 | 18.396 | 19.6 | 18.9 |
| IX 2003 | 13.95 | 15.15 | 14.53 |
| X 2003 | 11.2 | 10.3 | 9 |
| V 2004 | 10.7167 | 12.2 | 12.567 |
| VI 2004 | 14.55 | 15.7 | 16.367 |
| VII 2004 | 17.783 | 18.85 | 19.4 |
| VIII 2004 | 19.23 | 20 | 20.2 |
| IX 2004 | 15.65 | 15.15 | 13.967 |
| X 2004 |  |  |  |
| V 2005 | 7.463 | 10.1 | 11.4 |
| VI 2005 | 17.267 | 19.075 | 20.73 |
| VII 2005 | 18.6 | 19.925 | 19.83 |
| VIII 2005 | 18.43 | 18.95 | 18.8 |
| IX 2005 | 16.3 | 15.775 | 14.3 |
| X 2005 | 10.1 | 9.55 | 8.43 |
| V 2006 | 9.101587 | 11.35 | 14.8 |
| VI 2006 | 14.215 | 15.075 | 16.067 |
| VII 2006 | 17.841 | 18.7 | 21.1 |
| VIII 2006 | 18.6702 | 19.325 | 18.967 |
| IX 2006 | 16.925 | 16.325 | 15.23 |
| X 2006 | 14.49167 | 11.5 | 9.1 |
| V 2007 | 11.313 | 9.15 | 9.867 |
| VI 2007 | 16.8726 | 18.075 | 20.967 |
| VII 2007 | 17.603 | 18.5 | 19.267 |
| VIII 2007 | 19.525 | 20.625 | 21.43 |
| IX 2007 | 15.5583 | 15.95 | 15 |
| X 2007 | 11.419 | 12.125 | 10.767 |
| V 2008 | 11.514 | 13.05 | 14.13 |
| VI 2008 | 16.167 | 17.9 | 18.63 |
| VII 2008 | 18.867 | 20.275 | 21.767 |
| VIII 2008 | 20.2167 | 20.725 | 20.7 |
| IX 2008 | 14.367 | 14.275 | 12.567 |
| X 2008 | 11.1 | 10.5 | 10 |
| V 2009 | 10.7341 | 12.6 | 15 |
| VI 2009 | 15.1083 | 15.95 | 15.7 |
| VII 2009 | 18.9 |  | 21.3 |
| VIII 2009 | 18.5167 | 19 | 18.1 |
| IX 2009 | 16.483 | 19.125 | 16.93 |
| X 2009 | 10.5857 | 6.625 | 4.6 |
| V 2010 | 10.071 | 10.1 | 13.53 |
| VI 2010 | 14.87 | 16.075 | 17.63 |
| VII 2010 | 20.282 | 21.51875 | 22.467 |
| VIII 2010 | 21.83 |  | 25.0167 |
| IX 2010 | 15.7167 | 14.9 | 13.1 |
| X 2010 | 10.0488 | 10.9 | 9.4 |
| V 2011 | 10.8095 | 10.975 | 13.6 |
| VI 2011 | 15.45 | 19.35 | 21 |
| VII 2011 | 20.29357 | 21.025 | 22.67 |
| VIII 2011 | 19.067 | 21.5625 | 20.83 |
| IX 2011 | 16.02857 | 14.783 | 14.1167 |
| X 2011 | 11.75 | 8.5 | 6.2 |
| V 2012 | 11.217857 | 13.35 | 14.63 |
| VI 2012 | 14.645 | 17.25 | 15.867 |
| VII 2012 | 18.16428571 | 19.65 | 20.1 |
| VIII 2012 | 18.65714286 | 21.075 | 23.567 |
| IX 2012 | 16.967 | 17.875 | 17.067 |
| X 2012 | 13.35 | 12.5 | 13.3 |
| V 2013 | 11.25 | 13.6 | 17.36 |
| VI 2013 | 14.45 | 18.175 |  |
| VII 2013 | 17.03 | 20.075 |  |
| VIII 2013 | 18.2 | 20.45 |  |
| IX 2013 | 14.3 | 15 |  |
| X 2013 | 10 | 8.125 |  |
| V 2014 | 12.083 | 15.275 | 17.9 |
| VI 2014 | 15.483 | 17.1 | 16.167 |
| VII 2014 | 18.6167 | 19.775 | 19.967 |
| VIII 2014 | 20.925 | 21.9 | 20.53 |
| IX 2014 | 16.867 | 16.7 | 14.467 |
| X 2014 | 12.0167 | 9.775 | 8.7 |
| V 2015 | 10.55 | 12.75 | 14.4 |
| VI 2015 | 16.6 | 18.3 | 19.03 |
| VII 2015 | 17.8 | 19.8 | 19.03 |
| VIII 2015 | 20.75 | 20.8 | 20.867 |
| IX 2015 | 16.43 | 16 | 14.93 |
| X 2015 | 11.2167 | 8.725 | 7.43 |
| V 2016 | 12.5167 | 14.85 | 15 |
| VI 2016 | 15.3 | 16.825 | 16 |
| VII 2016 | 18.05 | 20.1 | 20.367 |
| VIII 2016 | 19.93 | 20.825 | 21.3 |
| IX 2016 | 17.45 | 16.2 | 15.067 |
| X 2016 | 8.8 | 9.85 | 9.03 |
| V 2017 | 7.5 | 10.6375 | 10.43 |
| VI 2017 | 15.75 | 16.6 | 18.67 |
| VII 2017 | 17.3167 | 18.75 | 19.8 |
| VIII 2017 | 20.63 | 21.325 | 21.83 |
| IX 2017 | 16.583 | 16.85 | 14.267 |
| X 2017 | 11.6 | 10.35 | 9.43 |
| V 2018 | 13.7167 | 14.6 | 17.1 |
| VI 2018 | 16.05 | 19.35 | 19.967 |
| VII 2018 | 17.45 | 19.8 | 20.13 |
| VIII 2018 | 20.6 | 21.3 | 21.067 |
| IX 2018 | 19.43 | 19.475 | 19.3 |
| X 2018 | 12.125 | 11.175 | 10.467 |
